# Analysis of the predictive value of heart rate variability analysis combined with SOFA and APACHE II scores for the 28-day mortality risk of patients in the emergency intensive care unit

**DOI:** 10.3389/fmed.2026.1832778

**Published:** 2026-05-12

**Authors:** Zhaolin Zhang, Hanlin Huang, Pengyi Pan, Longtao Zhang, Chen Zhang, Guoyu Lu

**Affiliations:** 1 Department of Emergency, The First Affiliated Hospital of Bengbu Medical University, Bengbu, Anhui, China; 2 Department of Emergency Surgery, Bengbu Third People's Hospital, Bengbu, Anhui, China

**Keywords:** autonomic nervous function, emergency intensive care, emergency treatment, heart rate variability, prognosis

## Abstract

**Objective:**

To explore the application value of heart rate variability (HRV) combined with SOFA score and APACHE II score in predicting the 28-day mortality risk of patients in the emergency intensive care unit (EICU), and to provide a basis for early clinical risk assessment.

**Method:**

A retrospective study was conducted on 115 patients admitted to the EICU of the First Affiliated Hospital of Bengbu Medical University. The patients were divided into the survival group (*n* = 75) and the death group (*n* = 40) based on their 28-day survival outcome. Demographic data, SOFA score, APACHE II score, and HRV-related indicators were collected. Univariate analysis, multivariate Logistic regression, Spearman correlation analysis, and receiver operating characteristic curve (ROC) analysis were used to evaluate the distribution characteristics and predictive efficacy of each indicator.

**Result:**

Compared with the survival group, the death group had significantly higher age, SOFA score, and APACHE II score (*p* < 0.05), and significantly lower HRV indicators such as PNS index and Mean RR (*p* < 0.01). Spearman analysis showed that SOFA score (*r* = 0.378) and APACHE II score (*r* = 0.456) were significantly positively correlated with death, while PNS index (*r* = −0.278) and Mean RR (*r* = −0.299) were negatively correlated with death (all *p* < 0.01). In the multivariate regression, only SDHR was an independent risk factor (OR = 3.211, *p* = 0.041). ROC curve analysis showed that the AUCs of HRV, SOFA score, and APACHE II score for predicting death were 0.737, 0.728, and 0.776, respectively. The AUC was 0.831 when the three were combined, indicating a significant improvement in predictive performance.

**Conclusion:**

Heart rate variability parameters can reflect the autonomic nerve function status of EICU patients. The combination of HRV with the SOFA score and APACHE II score system can improve the accuracy of predicting the 28-day mortality risk of EICU patients.

## 1 Introduction

Most patients admitted to the Emergency Intensive Care Unit (EICU) have complex and rapidly progressing critical conditions. Early and accurate identification of prognosis risks is crucial for guiding treatment strategies, optimizing resource allocation, and improving survival rates (1, 2). Currently, commonly used clinical scoring systems such as the Sequential Organ Failure Assessment (SOFA) score and the Acute Physiology and Chronic Health Evaluation II (APACHE II) score are widely applied in the assessment of critical patients’ conditions and prediction of mortality risks (3, 4). However, these scoring systems are mainly based on static physiological parameters and lack real-time reflection of the patients’ autonomic nervous system status. Heart rate variability (HRV), as a dynamic physiological indicator objectively reflecting the function of the autonomic nervous system (5), has attracted attention in critical care monitoring in recent years (6–9). Studies have shown that decreased HRV is closely related to inflammatory response (10, 11), organ dysfunction (12), and poor prognosis (7, 13), and can reflect changes in the balance between the sympathetic and parasympathetic systems (14), providing valuable information for assessing patients’ physiological reserves and stress responses. However, whether HRV can be combined with traditional scoring systems to enhance the identification of mortality risks in critically ill patients remains poorly studied.

This study aims to evaluate the predictive value of HRV combined with SOFA score and APACHE II score for 28-day mortality risk in EICU patients. By retrospectively analyzing multiple clinical parameters and HRV indicators, the study compares their independence and combined predictive ability, providing a basis for the application of HRV in the early prognosis assessment of critically ill patients.

## 2 Materials and methods

### 2.1 Research subjects

This study collected the clinical data of 115 emergency patients admitted to the emergency internal medicine department of the First Affiliated Hospital of Bengbu Medical University from August 2024 to November 2024. This study was approved by the Ethics Committee of Bengbu Medical University [No. 071, 2021]. A total of 115 EICU patients were included in the study, including 46 males and 69 females, with an average age of 71.5 years. The EICU where this study was conducted is an emergency intensive care unit in a tertiary hospital, mainly admitting critically ill patients from the emergency department, including those with septic shock, acute respiratory failure, cardiogenic shock, severe trauma and multiple organ dysfunction. The ward is equipped with continuous electrocardiogram (ECG) monitoring, hemodynamic monitoring and life support systems. All included patients completed the SOFA score, APACHE II score and ECG monitoring in the early stage of admission to the department, thus ensuring the standardization and consistency of data collection. Inclusion criteria: patients with complete general information, SOFA score and APACHE II score; patients with severe conditions requiring intensive supportive treatment; patients with complete ECG data and no special variations; exclusion criteria: patients with incomplete general and special information; patients with a history of malignant tumors; patients with a history of autoimmune diseases; patients without follow-up data, incomplete follow-up data or lost to follow-up.

### 2.2 Research methods

The general information of the patients was collected through the hospital medical record system, including: gender and age. All patients underwent SOFA score and APACHE II score assessment after admission, and their ECG data were collected and analyzed on the second day after admission to the EICU. All patients were followed up for 28 days. The patients were divided into the survival group and the death group based on their survival status, and the statistical differences in general information, HRV, SOFA score, and APACHE II score between the two groups were compared.

### 2.3 Data collection

All enrolled patients had their disease severity evaluated within the first 24 h after admission, which included: (1) SOFA score: This score assesses organ function from six aspects – respiratory, coagulation, liver, circulation, nervous system, and kidneys – to evaluate the degree of multiple organ dysfunction. (2) APACHE II score: This score comprehensively assesses patients based on acute physiological indicators, age, and chronic health conditions to predict the risk of death in critically ill patients. To minimize bias caused by dynamic changes in the condition, all scores were strictly calculated in accordance with international standards, and the worst physiological indicator values within the first 24 h after entering the EICU were uniformly used for calculation. The scoring process was independently completed by two senior attending physicians with experience in critical care medicine; when there were differences in the scores between the two, a third senior physician would review and make the final determination to enhance the consistency and reliability of the scores.

Within the first 24 h after the patient was admitted to the EICU, a 5-min high-sampling-rate ECG signal was continuously collected using a portable multi-parameter ECG monitor during the quiet morning period (08:00–10:00). The R waves (high-amplitude waves during the systolic phase of the heart) in the ECG were identified, and the time difference between adjacent R waves was calculated to obtain the raw RR interval sequence data. After the raw data was exported, Kubios HRV Premium 3.5 software was used for noise filtering, outlier detection, and other preprocessing steps. The software automatically removed artifacts and non-sinusoidal heartbeats to ensure the reliability of the data analysis. Only then could the calculation of heart rate variability research indicators proceed. The relevant indicators were calculated, including time-domain indicators (Mean RR, SDNN, RMSSD, SDHR), nonlinear indicators (DCmod, ACmod), and comprehensive indices (PNS index, SNS index, Stress index). Among them, the calculation methods for each of the HRV data collected are as follows (Table 1).

**Table 1:** HRV parameters and calculation methods.

HRV parameters	Calculation methods
Mean RR	Average of all normal-to-normal (NN) intervals
SDNN	Standard deviation of NN intervals (overall HRV)
RMSSD	Root mean square of successive NN differences (parasympathetic activity)
SDHR	Standard deviation of heart rate values
PNS index/SNS index	Composite indices reflecting parasympathetic and sympathetic activity
DCmod/ACmod	Derived using phase-rectified signal averaging (PRSA), representing deceleration and acceleration capacities of heart rate, respectively

### 2.4 Statistical methods

Statistical analysis was conducted using SPSS 25.0. Data that followed a normal distribution were expressed as mean ± standard deviation (Mean ± SD), and comparisons between groups were performed using the independent sample t-test; non-normal distribution data were expressed as median (interquartile range) [*M* (IQR)], and group comparisons used non-parametric tests. Categorical variables were expressed as frequency (percentage), and group comparisons used the chi-square test (*χ*^2^ test). Multivariate Logistic regression was used to evaluate the independent predictors of patient death. The PNS index, SNS index and other HRV-related indicators were incorporated into the binary Logistic regression model, and the “predicted probability” output by the model was used as the “HRV indicator” in the ROC analysis. Meanwhile, the Hosmer–Lemeshow goodness-of-fit test was adopted to evaluate the model, in order to understand the model’s fitting degree. The predictive ability of SOFA score, APACHE II score, HRV indicators, and their combined model for 28-day mortality was evaluated through the receiver operating characteristic (ROC) curve. Spearman rank correlation analysis was used to explore the correlation between clinical indicators and the 28-day mortality outcome. A *p* value less than 0.05 was considered significant.

## 3 Results

A total of 115 patients were included in this study, among which 75 were in the survival group and 40 in the death group (Table 2). There was no significant difference in gender composition between the two groups (male/female: 36%/64% vs. 47%/53%, *p* = 0.231). The age of patients in the death group was significantly higher than that in the survival group [median 73.5 years (IQR: 63–82) vs. 69 years (IQR: 54–77), *p* = 0.036]. In terms of disease severity scores, the SOFA score of the death group was significantly higher than that of the survival group [7 (5–9) vs. 4 (2–7), *p* < 0.001], and the APACHE II score was also significantly increased (21.63 ± 5.15 vs. 14.96 ± 6.99, *p* < 0.001). In terms of HRV related indicators, the PNS index of the death group was significantly lower than that of the survival group (−2.21 ± 0.98 vs. −1.54 ± 1.07, *p* = 0.001), and the SNS index also increased [9.09 (6.25–11.72) vs. 6.24 (4.05–10.26), *p* = 0.008]. The Stress index in the death group also showed an increasing trend, but the difference was not statistically significant (*p* = 0.075). In the time domain analysis, the Mean RR of the death group was significantly lower than that of the survival group (636.19 ± 164.17 ms vs. 741.33 ± 162.61 ms, *p* = 0.001). While other time domain indicators such as SDNN, SDHR, and RMSSD showed no statistically significant differences between the two groups (*p* > 0.05). In the nonlinear HRV indicators, the ACmod in the death group was significantly higher than that in the survival group [−8.96 (4.54–14.69) ms vs. −11.70 (6.55–20.21) ms, *p* = 0.049], and DCmod showed a downward trend in the two groups, but did not reach statistical significance (*p* = 0.063).

**Table 2:** Baseline data and univariate analysis table.

Group	Survival (n)	Death (n)	Total (n)	*P*-value
Patients (*n*)	75	40	115	–
Sex	75[100]	40[100]	115[100]	0.231
Male (*n*)	27[36]	19[47]	46[40]	
Female (*n*)	48[64]	21[53]	69[60]	
Age (years)	69(54–77)	73.5(63–82)	71.5(56–79)	0.036
SOFA score	4(2–7)	7(5–9)	6(3–7)	0.000
APACHE II score	14.96 ± 6.99	21.63 ± 5.15	17.26 ± 7.11	0.000
PNS index	-1.54 ± 1.07	−0.21 ± 0.98	−1.77 ± 1.08	0.001
SNS index	6.24(4.05–10.26)	9.09(6.25–11.72)	7.05(4.76–11.45)	0.008
Stress index	42.18(31.80–59.47)	55.36(37.16–67.00)	48.21(33.17–63.46)	0.075
Mean RR (ms)	741.33 ± 162.61	636.19 ± 164.17	702.77 ± 170.66	0.001
SDNN (ms)	10.66(6.06–16.79)	8.14(4.97–13.76)	8.81(5.96–16.04)	0.184
SDHR (bpm)	1.18(0.72–1.63)	1.09(0.82–2.09)	1.17(0.80–1.72)	0.948
RMSSD (ms)	11.00(6.19–18.31)	8.44(4.77–13.76)	9.86(6.09–17.07)	0.103
DCmod (ms)	12.04(7.00–20.04)	8.76(5.68–14.78)	10.25(6.20–19.04)	0.063
ACmod (ms)	−11.7(20.21 ± 6.55)	−8.96(14.69 ± 4.54)	−10.48(19.55 ± 6.33)	0.049

To further explore the independent risk factors influencing the prognosis of patients, a multivariate Logistic regression analysis was conducted on multiple indicators including general demographic characteristics, disease severity scores, and HRV-related parameters. The results (Table 3) showed that SDHR (standard deviation of heart rate) was the only significant factor, with a regression coefficient B of 1.167, *p* = 0.041, and the corresponding OR of 3.211 (95% CI: 1.052–9.804), suggesting that an increase in SDHR was associated with an increased risk of patient death. The remaining variables, including gender (*p* = 0.314), age (*p* = 0.337), SOFA score (*p* = 0.153), APACHE II score (*p* = 0.071), PNS index (*p* = 0.243), SNS index (*p* = 0.083), and Stress index (*p* = 0.067), did not reach statistical significance after multivariate correction (*p* > 0.05). Additionally, time-domain indicators (Mean RR, SDNN, and RMSSD) and nonlinear indicators (DCmod, ACmod) did not show significant independent predictive efficacy (*p* > 0.05).

**Table 3:** Multivariate logistic regression analysis of patient prognosis.

Variable	*B*	SE(b)	Wald *χ*^2^	*p*-value	OR	95%CI
Sex	−0.52	0.516	1.016	0.314	0.595	0.216–1.634
Age (years)	0.019	0.02	0.923	0.337	1.019	0.981–1.059
SOFA score	0.154	0.108	2.044	0.153	1.167	0.944–1.441
APACHE II score	0.092	0.051	3.254	0.071	1.097	0.992–1.212
PNS index	5.584	4.787	1.361	0.243	266.109	0.022–3158263.174
SNS index	1.277	0.736	3.012	0.083	3.584	0.848–15.152
Stress index	−0.241	0.132	3.347	0.067	0.786	0.607–1.017
Mean RR	−0.015	0.018	0.636	0.425	0.986	0.951–1.021
SDNN	0.006	0.123	0.002	0.961	1.006	0.791–1.28
SDHR	1.167	0.569	4.196	0.041	3.211	1.052–9.804
RMSSD	−0.284	0.343	0.687	0.407	0.753	0.384–1.474
DCmod	−0.145	0.162	0.81	0.368	0.865	0.63–1.187
ACmod	−0.027	0.114	0.056	0.814	0.973	0.778–1.217

**Figure 1:**
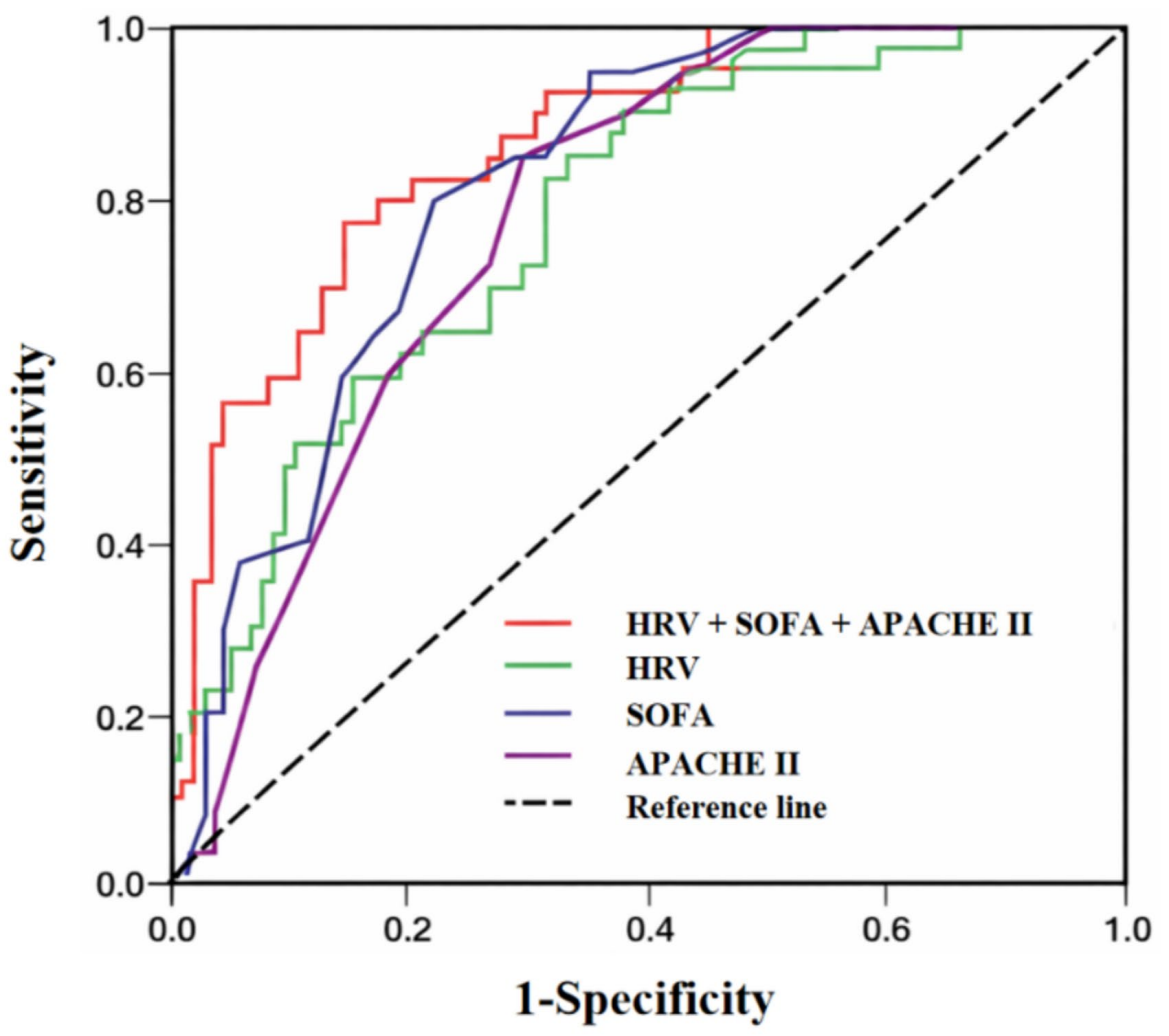
ROC curves of HRV, SOFA score, APACHE II score, and the combination of the three for predicting patient mortality.

To evaluate the predictive ability of HRV indicators, SOFA score, APACHE II score, and their combined model for the risk of patient mortality, corresponding ROC curves were plotted (Figure 1). The results showed that the combined model of HRV, SOFA score, and APACHE II score (red line) had the best predictive efficacy, with the curve distributed most widely in the upper left corner, suggesting that its sensitivity and specificity were superior to those of individual indicators. In contrast, the predictive ability of HRV (green line), SOFA score (blue line), and APACHE II score (purple line) alone was weak, and the curves shifted downward significantly. The AUC (area under the curve) of the combined model was higher than that of the single-score model, suggesting that HRV, as a dynamic reflection of physiological status, combined with disease severity scores, can more effectively predict the adverse prognosis of patients. Further evaluation of the predictive ability of each variable for the risk of patient mortality showed (Table 4): In the single indicators, the AUC of APACHE II score was the highest (0.776, 95% CI: 0.693–0.860), with a good sensitivity (0.80) and specificity (0.667) when the cutoff value was 17.5, and the Youden index was 0.467. The AUC of SOFA score was 0.728 (95% CI: 0.637–0.819), with a sensitivity and specificity of 0.85 and 0.56, respectively, when the cutoff value was 4.5, and the Youden index was 0.41. The AUC of HRV was 0.737 (95% CI: 0.645–0.830), with a sensitivity of 0.60 and specificity of 0.76 when the cutoff value was 0.3817, and the Youden index was 0.36. The AUC of the combined model (HRV + SOFA + APACHE II) reached 0.831 (95% CI: 0.754–0.907), higher than any single indicator, indicating better comprehensive predictive ability. This combined model had a sensitivity and specificity of 0.775 and 0.773, respectively, when the cutoff value was 0.354, with the highest Youden index (0.548), suggesting that it has higher discriminative efficacy in the identification of mortality risk.

**Table 4:** Predictive value of independent risk factors associated with patient mortality.

Variable	AUC	95%CL	Cutoff value	Sensitivity	Specificity	Youden index	*P*-value
HRV	0.737	0.645–0.83	0.3816598	0.6	0.76	0.36	0.000
SOFA score	0.728	0.637–0.819	4.5	0.85	0.56	0.41	0.000
APACHE II score	0.776	0.693–0.86	17.5	0.8	0.667	0.467	0.000
HRV + SOFA+APACHE II	0.831	0.754–0.907	0.3543276	0.775	0.773	0.548	0.000

Spearman correlation analysis was used to evaluate the correlations between various indicators and the patient’s mortality outcome. The analysis results (Table 5) showed that age, SOFA score, APACHE II score, SNS index, and ACmod were all positively correlated with the mortality outcome. Among them: the APACHE II score (*r* = 0.456, *p* < 0.001) and the SOFA score (*r* = 0.378, *p* < 0.001) were moderately positively correlated with the risk of death; age (*r* = 0.196, *p* = 0.035) and SNS index (*r* = 0.249, *p* = 0.007) were slightly positively correlated with the risk of death; the nonlinear HRV index ACmod was also slightly positively correlated with death (*r* = 0.185, *p* = 0.048). On the contrary, PNS index (*r* = −0.278, *p* = 0.003) and Mean RR (*r* = −0.299, *p* = 0.001) were negatively correlated with the risk of death, suggesting that the stronger the parasympathetic nerve function or the longer the heart rate interval, the lower the risk of death. However, gender, SDNN, SDHR, RMSSD, Stress index, and DCmod did not show statistically significant correlations with the mortality outcome (*p* > 0.05).

**Table 5:** Correlation analysis of factors contributing to patient deaths.

Variable	Follow-up outcomes	
	Spearman’s *ρ*	*P*-value
Sex	−0.112	0.234
Age (years)	0.196	0.035
SOFA score	0.378	0.000
APACHE II score	0.456	0.000
PNS index	−0.278	0.003
SNS index	0.249	0.007
Stress index	0.167	0.075
Mean RR	−0.299	0.001
SDNN	−0.124	0.186
SDHR	0.006	0.949
RMSSD	−0.153	0.103
DCmod	−0.174	0.063
ACmod	0.185	0.048

## 4 Discussion

In this study, by comparing the baseline characteristics and HRV parameters of patients who survived and those who died in the emergency intensive care unit, it was found that the SOFA score and APACHE II score showed significant differences among different prognostic groups, indicating their good application value in risk stratification of critically ill patients (15). The baseline data analysis results showed that the median SOFA score of the death group was 7 (IQR: 5–9), significantly higher than that of the survival group’s 4 (IQR: 2–7) (*p* < 0.001); the APACHE II score of the death group was 21.63 ± 5.15, also significantly higher than that of the survival group’s 14.96 ± 6.99 (*p* < 0.001). At the same time, in the death patients, the PNS index decreased, the SNS index increased, and the Mean RR shortened, reflecting the more prominent autonomic nerve dysfunction in death patients, suggesting that autonomic nerve dysfunction may play an important role in the deterioration of the condition. This might be related to the amplification of inflammation and hemodynamic instability. These result are consistent with previous studies, that is, excessive activation of the sympathetic nerve and inhibition of the vagus nerve activity are typical manifestations of poor prognosis in critically ill patients (16–20). The abnormality of ACmod in deceased patients further reflects the decline in the ability to accelerate heart rate and the impairment of the stress response.

Although some HRV indicators such as Stress index, DCmod, and RMSSD did not reach a significant level statistically, the overall trend also supported this physiological mechanism. In the multivariate Logistic regression analysis, SDHR was selected as an independent predictor of death (OR = 3.211, *p* = 0.041). SDHR, as an indicator measuring the amplitude of HRV, is often considered clinically to be closely related to the sympathetic-vagal regulatory ability, and its increase may represent instability in heart rate control, suggesting a decline in the regulatory ability of the autonomic nerve, which is a signal of deterioration in the patient’s condition (20–24). For critically ill patients, this “instability” may indicate that although the body is still attempting to maintain the balance of circulation and metabolism under high stress, its autonomous regulatory capacity has significantly declined. The compensatory mechanisms no longer show orderly and controllable fluctuations, but instead transform into chaotic, inefficient or even imbalanced response patterns. This result suggests that after adjusting for disease severity and demographic variables, SDHR can be used as an important monitoring indicator for evaluating recovery in patients in the emergency intensive care unit. The ROC curve analysis results showed that HRV, SOFA score, and APACHE II score all had certain predictive ability for patient recovery (AUCs were 0.737, 0.728, and 0.776, respectively). The SOFA score assesses the multi-organ function status and is suitable for dynamic monitoring (25–28); while the APACHE II score integrates acute physiological indicators, age, and chronic health scores, reflecting a more comprehensive overall status, and is more sensitive in predicting the risk of death (29–33). The results of this study indicate that the model constructed by HRV combined with SOFA score and APACHE II score shows better predictive ability, with an AUC of 0.831, significantly improving the prediction accuracy. This suggests that HRV indicators, as a dynamic reflection of patient physiological fluctuations, can provide additional supplementary information on top of traditional scoring systems, especially helping to improve the specificity and comprehensive discrimination ability of the model, indicating that the combined prediction has more application value in clinical risk assessment (6, 13, 34–36). HRV can be used as a complementary indicator to SOFA score and APACHE II score, providing important basis for early risk stratification and treatment decisions for patients.

The results of the correlation analysis further verified the above findings. The results showed that the SOFA score was moderately positively correlated with patient mortality (*r* = 0.378, *p* < 0.001), and the APACHE II score was strongly positively correlated with mortality (*r* = 0.456, *p* < 0.001). This indicates that the increase in the numerical values of both scores has a consistent trend with the increased risk of death, and the correlation of APACHE II score is stronger, suggesting that it may be more sensitive in reflecting systemic physiological dysfunction and the severity of the disease. Among the HRV-related parameters, the PNS index (*r* = −0.278, *p* = 0.003), Mean RR (*r* = −0.299, *p* = 0.001) were negatively correlated with mortality, while the SNS index (*r* = 0.249, *p* = 0.007) and ACmod (*r* = 0.185, *p* = 0.048) were positively correlated with mortality. This indicates that the dysfunction of the autonomic nervous system is highly correlated with the outcome of death: enhanced sympathetic activity, vagal inhibition, and shortened heart rate intervals may be important physiological bases for the deterioration of prognosis (37, 38). Although Stress index, DCmod, etc. were not significantly correlated, they also showed a similar trend, suggesting that these indicators may exhibit statistical significance in larger samples.

In conclusion, HRV can be regarded as a dynamic indicator reflecting the autonomic nerve regulation status of patients. Its advantages in the combined scoring model are obvious, providing important supplementary value for the early identification of the prognosis risk of critically ill patients. The combination of HRV with SOFA and APACHE II scores can effectively improve the predictive accuracy of 28-day mortality risk in EICU patients, providing important supplementary evidence for the early identification of high-risk patients and the optimization of intervention measures in clinical practice. As a quantifiable, dynamic, and non-invasive physiological indicator, HRV is expected to be applied in the early risk stratification and dynamic prognosis assessment of critically ill patients, improving the efficiency and timeliness of clinical identification of high-risk populations. Although the results of this study have certain clinical significance, several limitations still exist. Firstly, this study is a single-center retrospective study with a relatively limited sample size, which may affect the generalizability and statistical power of the results. Secondly, this study is based only on short-term HRV analysis and did not conduct continuous dynamic monitoring, which may not fully reflect the dynamic changes in the autonomic nerve function of patients during disease progression. In addition, in the multivariate Logistic regression analysis, some variables did not reach statistical significance, which may be related to the collinearity among variables or the limited statistical power due to the insufficient sample size, thereby affecting the determination of independent effects. Future studies should conduct large-sample, multi-center prospective studies and combine dynamic HRV monitoring to further verify the stability and clinical application value of the conclusions of this study. Future studies should be conducted with larger sample sizes and multi-center prospective designs to further verify its clinical practicability and promotion value.

## 5 Conclusion

The SOFA score, APACHE II score, and parameters related to HRV are closely associated with the 28-day mortality risk of patients in the EICU. The combined diagnosis of these three factors can significantly improve the accuracy of the prediction model.

## Data Availability

The original contributions presented in the study are included in the article/supplementary material, further inquiries can be directed to the corresponding author.
